# Retrospective Preliminary Assessment of Routine Follow-Up Low-Field Magnetic Resonance Imaging in Dogs Presumptively Diagnosed With Discospondylitis

**DOI:** 10.3389/fvets.2022.880038

**Published:** 2022-05-18

**Authors:** Maria Ines de Freitas, Enzo Vettorato, Elena Scarpante, Giunio Bruto Cherubini, Abby Caine

**Affiliations:** Dick White Referrals, Linnaeus Veterinary Ltd, Cambridgeshire, United Kingdom

**Keywords:** discospondylitis, MRI, vertebral endplate, dog, spine, intervertebral disc

## Abstract

**Background:**

The usefulness of routine follow-up Magnetic Resonance Imaging (MRI-2) in asymptomatic dogs treated for discospondylitis is unknown.

**Methods:**

This cross-sectional retrospective study investigated the features of MRI-2 in a heterogeneous group of dogs treated for discospondylitis, and if these were associated with the presence or absence of clinical signs. After comparing initial MRI (MRI-1) and MRI-2, an observer, blinded to the dog's clinical signs, described the MRI-2 findings. The study population was then divided into symptomatic or asymptomatic at the time of MRI-2. Two separate observers subjectively classified the discospondylitis as active or inactive. Repeatability and interobserver agreement were evaluated.

**Results:**

A total of 25 dogs were included. At the time of MRI-2 16 (64%) dogs were asymptomatic and 9 (36%) were symptomatic. Based on MRI-2, 20 (80%) and 18 (72%) out of 25 dogs were considered to have active discospondylitis by the first and second observers, respectively. Interobserver agreement was moderate. No MRI-2 features were associated with the clinical status. The subjective classification of inactive discospondylitis was significantly associated with asymptomatic clinical status, but the classification of active discospondylitis was evenly distributed between groups.

**Conclusion:**

This study did not identify a meaningful association between the clinical status of dogs treated for presumptive discospondylitis and MRI-2 results. There were no specific MRI-2 features which were associated with the clinical status.

## Introduction

Discospondylitis is an infection of one, or more, intervertebral disc (IVD) spaces and adjacent vertebral endplates (1). The infection may also affect the vertebral bodies and surrounding soft tissues or extend into the vertebral canal and cause epidural empyema (2). Diagnosis of discospondylitis may be challenging as clinical signs are often unspecific (1). Magnetic resonance imaging (MRI) is the most sensitive and specific modality for the detection of infections of the vertebral column in human patients (3, 4) and its use in dogs with discospondylitis has been well documented (5–8).

Treatment for discospondylitis generally consists of administration of antibiotic(s), but surgical intervention may be necessary in some dogs (2, 9, 10). While various antibiotic protocols have been proposed, the appropriate length of the treatment is unknown (1, 11, 12). Relapse of the condition is likely to occur if antibiotics are discontinued prematurely (13). Furthermore, clinical signs and diagnostic imaging findings are often incongruent during disease progression, complicating the decision regarding when to interrupt treatment. Radiographic deterioration of discospondylitis was reported despite improvement of clinical signs in dogs (14).

While MRI is more sensitive than radiography for diagnosing discospondylitis (5, 15, 16), the value of routine follow-up MRI (MRI-2) to assist clinical decision making regarding the appropriate time point of antibiotic therapy discontinuation in asymptomatic dogs has not been evaluated. In human medicine, the usefulness of routine MRI-2 in asymptomatic patients is questionable with multiple studies reporting progressive imaging deterioration despite successful clinical response to the treatment (17–19). In fact, the clinical practice guidelines of the Infectious Disease Society of America do not recommend follow-up MRI in patients with a favorable clinical response to therapy in vertebral osteomyelitis (20).

The aim of our study is to describe MRI-2 features in a heterogeneous group of dogs presumptively diagnosed with discospondylitis and to investigate if there is an association between the MRI-2 features and the clinical status. We hypothesize that MRI-2 findings will be unspecific and not associated with the clinical status.

## Materials and Methods

The medical records of dogs diagnosed with and treated for discospondylitis by an ECVN board-certified neurologist, or ECVN resident, at Dick White Referrals from 2010 to 2019 were retrieved from the electronic database. Signed owner consent for the use of clinical information was obtained at the time of the animal admission to the hospital. No ethical approval was obtained due to the retrospective nature of this cross-sectional study, and prior acquisition of written owner consent for patient data to be included in scientific studies.

Cases were immediately excluded if: (1) clinical records were not complete up to the time of MRI-2; (2) antibiotics were not administered as part of the treatment; (3) MRI-2 of the affected vertebral column region was not performed at least 28 days from diagnosis.

Of the remaining cases, the MRI study obtained on initial presentation (MRI-1) was reviewed by an ECVDI-certified veterinary radiologist (AC) unaware of the dog's clinical signs. Only cases that fulfilled the criteria for presumptive diagnosis of discospondylitis were included in the study. Specifically, involvement of the intervertebral disc and adjacent vertebral endplates, a short-tau inversion recovery (STIR) hyperintense signal and/or contrast enhancement of the paravertebral soft-tissues and at least one of the following features: presence of a STIR hyperintense signal and/or contrast enhancement of the IVD, STIR hyperintense signal or T2-Weighted (T2w) hypointense or hyperintense signal of the adjacent endplate(s) (6, 7, 21).

Of the final population of dogs included in the study, signalment and clinical information (Table 1), culture results from urine, blood, or affected IVD samples were recorded. The IVD samples were collected by ultrasound-guided percutaneous fine-needle-aspiration or intraoperatively. Treatment following diagnosis was recorded as medical, if only antibiotic therapy and analgesic drugs were administered; or surgical, if surgery preceded the antibiotic therapy. The type of medical treatment following MRI-1, including antibiotic and/or analgesic therapy was recorded, as well as whether antibiotic therapy was continued following MRI-2. The time elapsed between MRI-1 and MRI-2 was also recorded.

**Table 1:** Demographic information and clinical data of 25 dogs with discospondylitis included in this study.

**Demographic information and clinical data**	
Breeds (*n*)	
- Labrador Retriever - Springer Spaniel - German Shepherd Dog - Basset hound - Beagle - Boxer - Crossbreed - Miniature Dachshund - English Bull Terrier - Hungarian Vizsla - Siberian Husky - Rhodesian Ridgeback - West Highland White Terrier	8 4 3 1 1 1 1 1 1 1 1 1 1
Sex (*n*)	
- Female entire-Female neutered - Male entire-Male neutered	4–6 13–2
Clinical signs (*n*)	
- Spinal pain - Paresis - Lameness	24 10 1
Age (months) [mean (± standard deviation)]	78 (±23)
Weight (kg) [mean (± standard deviation)]	29.2 (±10.3)
Duration of clinical signs (days) [median (95% confidence intervals)]	30 (7–75)
Cause of discospondylitis (*n*)	
- Natural - Post-surgical	20 5
Time from surgery to discospondylitis (days) [median (95% confidence intervals)]	75 (9–102)
Affected disc spaces (*n*)	
- L7-S1 - L8-S1 - T12-13 - L2-3 - L3-4 - C6-7 - L1-2 - L1-3 - L5-6 - L6-7	13 1 2 2 2 1 1 1 1 1

*Data are reported as mean (± standard deviation) or median (95% confidence intervals). n, number of dogs; C, cervical; T, thoracic; L, lumbar; S, sacral*.

The cases were divided into two groups based on the clinical status at the time of MRI-2. Dogs were assigned to the “asymptomatic” group if clinical signs had resolved and MRI-2 was performed only as part of a re-examination, or to the “symptomatic” group if response to the treatment was unsatisfactory due to either clinical deterioration or failure to clinically improve.

Both MRI-1 and MRI-2 were performed under general anesthesia using a low-field, 0.4 T, permanent, open magnet (Aperto Lucent, Hitachi Medical Corporation, Tokyo, Japan) to include the portion of the spinal cord indicated by the clinical neuro-localization on MRI-1, and to include the previously diagnosed discospondylitis site on MRI-2. Dogs were positioned in dorsal recumbency with the pelvic limbs in a neutral position. The first observer (AC), blinded to the dog's clinical status, reviewed the acquired images and documented the location of affected disc space and presence or absence of specific features, all of which are summarized in Table 2.

**Table 2:** Type of treatment between MRI-1 (magnetic resonance imaging at initial presentation) and MRI-2 (follow-up), defined subjectively as active or inactive discospondylitis after MRI-2 in symptomatic and asymptomatic dogs.

	**Symptomatic (*n* = 9)**	**Asymptomatic (*n* = 16)**	***p*-value**	**OR (95%CI)**
Treatment	5 Medical 4 Surgical	8 Medical 8 Surgical	0.99	
1^st^assessment of discospondylitis on MRI-2 (*n*)	8 Active 1 Inactive	12 Active 4 Inactive	0.005*	12 (1.77–512.97)
2^nd^ assessment of discospondylitis on MRI-2 (*n*)	7 Active 2 Inactive	11 Active 5 Inactive	0.026	5.5 (1.20–51.06)

*n, number of dogs; OR, odd ratio; CI, 95% confidence intervals*.

Two observers (AC and ES), unaware of the dog's clinical status, compared MRI-1 and MRI-2 and subjectively classified each case as active or inactive discospondylitis, based on the overall interpretation of the MRI-2 findings. To assess intra-observer repeatability of this subjective assessment, the images were re-assessed 2 months later by the first observer (AC), and the scores repeated.

Whilst no single feature independently led to a case being assigned “active” or “inactive”, the lack of regional lymphadenomegaly; the lack of STIR hyperintense signal and/or contrast enhancement of the perilesional soft tissues, beyond the tissues dissected during the previous surgical approach; well defined endplates with no-to-mild STIR hyperintensity; lack of STIR hyperintense epidural material; no-to-mild only enhancement of the affected vertebrae; T2-W and STIR hypointense intravertebral discs compared to spinal cord were findings considered to be suggestive of inactive disease. In contrast, regional lymphadenomegaly; STIR hyperintensity and/or contrast enhancement of the perilesional soft tissues, beyond the tissues dissected during the previous surgical approach; ill-defined and effaced endplates with strong STIR hyperintensity; STIR hyperintense epidural material; strong contrast enhancement of the endplates and bodies of the affected vertebrae were features which were considered to be suggestive of active disease (6–8, 21). In cases where the features suggestive of active and inactive overlapped, the classification was awarded based on observer's subjective assessment.

### Statistical Analysis

The distribution of continuous variables was assessed using D'Agostino and Pearson test and results are reported as mean ± standard deviation or median [95% confidence intervals (CI)], accordingly. Mann-Whitney U test was used to compare the time between MRI-1 and MRI-2 between symptomatic and asymptomatic groups.

McNemar test was used to analyze paired ordinal variables and to assess the presence of systematic difference between: (1) specific features detected on MRI-1 and MRI-2; (2) presence of active discospondylitis or inactive discospondylitis on MRI-2 between symptomatic and asymptomatic groups; (3) repeatability of identifying active discospondylitis or inactive discospondylitis on MRI-2.

The Cohen's Kappa (k) coefficient was calculated to measure inter-observer agreement.

Fisher's exact test was used to analyze unpaired ordinal variables: (1) type of treatment (medical or surgical) between symptomatic and asymptomatic groups; (2) type of treatment and active or inactive discospondylitis on MRI-2.

A p < 0.05 was considered statistically significant. Odd ratio (OR) and 95% CI, sensitivity and specificity are reported when appropriate.

To study if any of the MRI-2 features were associated with the presence of clinical signs a backward stepwise regression was performed. Variables with variance inflation factor (VIF) > 5 were excluded because of multicollinearity. Hosmer-Lemshow test and Likelyhood ratio test were used to confirm the good fitness of the model used.

## Results

**Figure 1:**
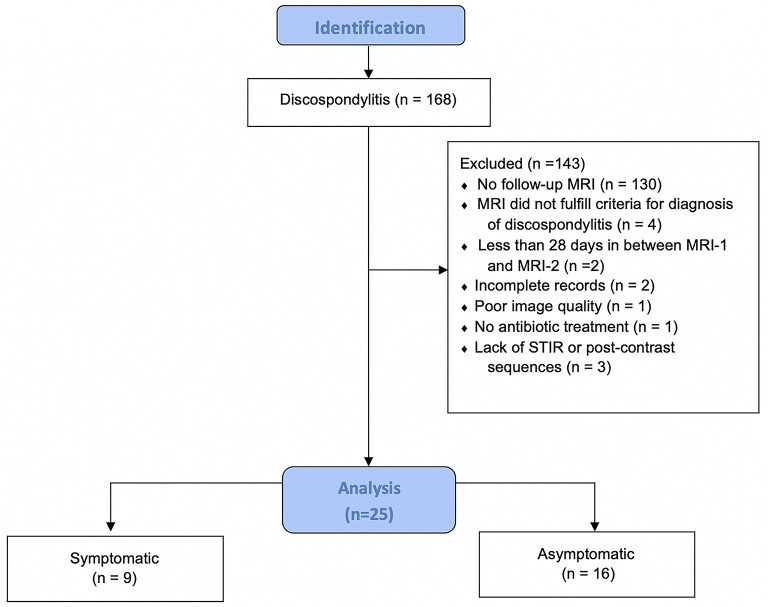
Flow diagram illustrating the inclusion and distribution of dogs in this study. MRI, magnetic resonance imaging; MRI-2, follow-up MRI; STIR, short-tau inversion recovery; *n*, number of dogs.

A total of 168 dogs presumptively diagnosed with discospondylitis were initially identified, but only 25 fulfilled the inclusion criteria (Figure 1).

**Figure 2:**
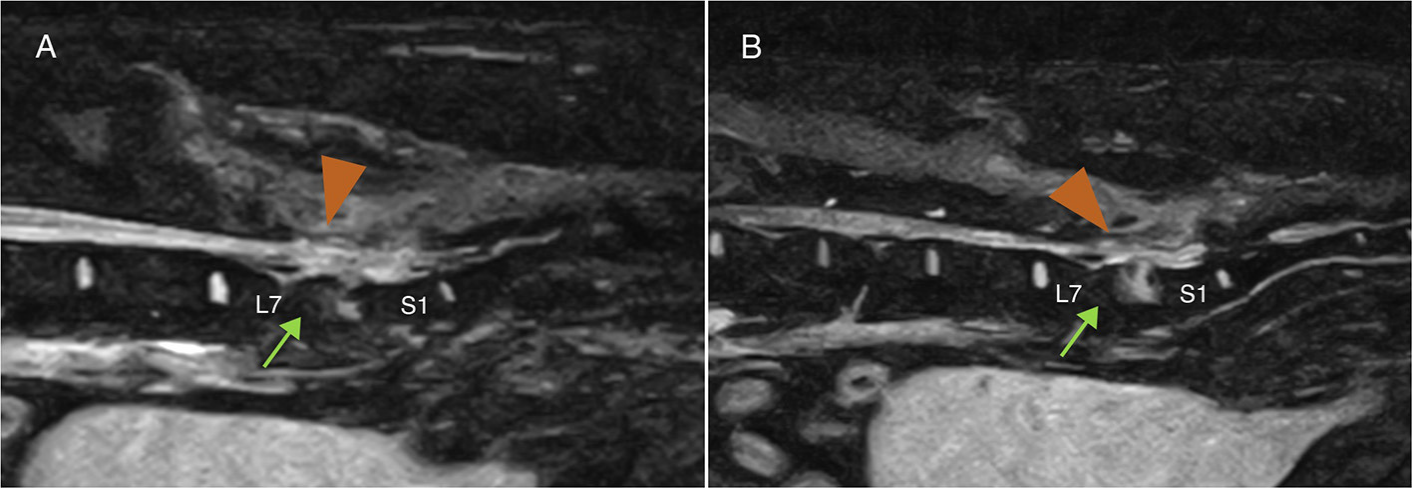
Midline **(A)** and 4 mm from midline **(B)** MRI-1 short-tau inversion recovery (STIR) sagittal images of a dog that developed discospondylitis post-operatively, 4 months following dorsal laminectomy and IVD annulectomy. The diagnosis was based on the combination of severe and progressive clinical signs, lack of significant persistent IVD protrusion causing neural tissue compression and changes affecting the epidural space (arrow-head) and the vertebral body (arrow), and erosion of the end plates.

**Figure 3:**
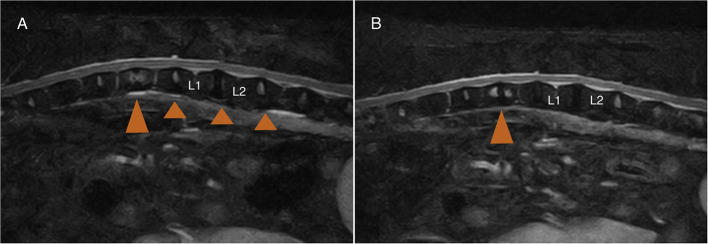
MRI-1 **(A)** 2 months post-surgery, and MRI-2 **(B)** 10 months post-surgery of a dog that underwent L1-L2 left hemilaminectomy and T12-L3 IVD fenestrations. The dog developed marked spinal pain and on MRI-1 **(A)** there was no evidence of neural tissue compression at the previous IVD extrusion site (L1-L2), and evidence of T12-T13 discospondylitis (large arrowhead) corresponding to a previously fenestrated disc site. Note the lack of STIR abnormalities in the other fenestrated discs (small arrowhead).

**Figure 4:**
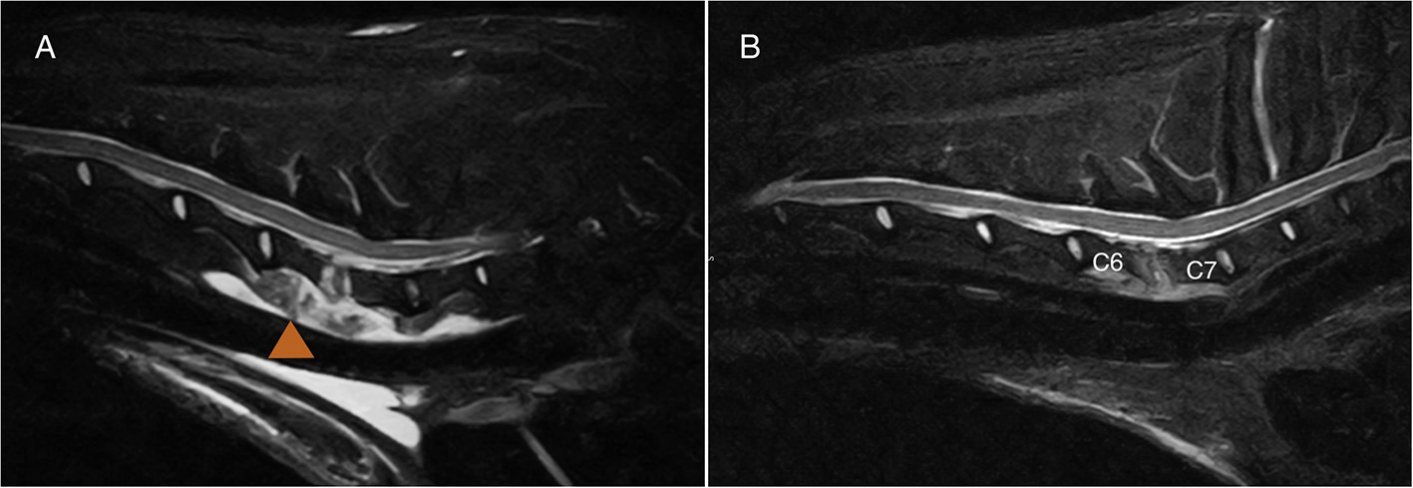
MRI-1 **(A)** and MRI-2 **(B)** midline sagittal short-tau inversion recovery (STIR) images of the case diagnosed with C6-C7 discospondylitis following ventral slot. One month separates MRI-1 and MRI-2. Note the extensive soft tissue changes (arrowhead) which my be associated with surgical exposure. The degree of effacement and STIR hyperintensity affecting the endplates, as well as the STIR hyperintense material in the epidural space were considered beyond what is expected following surgery. The dog had cervical spinal pain despite lack of obvious neural tissue compression. The dog was “symptomatic” at the time of MRI-2 and both observers classified the case as “active.”

Demographic and clinical data of the population of dogs included in this study are summarized in Table 1. Spinal hyperesthesia and paresis were reported in 96 and 40% of the dogs, respectively. Clinical signs were present for a median of 30 days before hospital admission. Discospondylitis was naturally occurring in 20/25 (80%) cases and developed after spinal surgery to address spinal cord compression secondary to intervertebral disc disease in 5/25 (20%) cases: 2 dogs following lumbosacral (LS) dorsal laminectomy, annulectomy (1/2) and IVD fenestration (1/2); 2 dogs following IVD fenestration performed at the time of thoracolumbar hemilaminectomy; and 1 dog following a cervical ventral slot. Time between surgery and development of discospondylitis was 9, 30, 75, 93, and 102 days. Dogs who underwent L7-S1 dorsal laminectomy (2/25) were diagnosed with discospondylitis based on clinical deterioration despite lack of neural tissue compression, presence of STIR hyperintense and/or enhancing soft tissues surrounding to the affected IVD, as well as abnormal IVD space and adjacent vertebral bodies (Figure 2). Dogs diagnosed with discospondylitis following thoracolumbar IVD fenestration (2/25) were diagnosed with discospondylitis based on marked IVD and endplate changes, and absence of similar changes in the remainder of the fenestrated discs (Figure 3). The dog that underwent ventral slot was subsequently diagnosed with discospondylitis based on severe endplate changes, characterized by strong STIR hyperintense signal which extended within the vertebral body and heterogenous, ill-defined endplate margins, as well as presence of STIR hyperintense epidural material and lack of neural tissue compression to explain the clinical deterioration (Figure 4).

In 56% of the dogs, discospondylitis affected the LS intervertebral disc. Treatment between MRI-1 and MRI-2 was medical in 13 (52%) dogs and surgical in 12 (48%) dogs. Surgical treatment was pursued at the clinician's discretion to address spinal cord compression secondary to disc extrusion or empyema (10/12), and/or curettage and acquisition of samples for culture (10/12). Surgical treatment consisted of: C6-C7 ventral slot revision for curettage and sample collection in 1/12 dogs; T12-T13 hemilaminectomy, spinal cord decompression and IVD fenestration in 1/12 dogs; L2-L3 IVD fenestration and curettage in 1/12 dogs; L3-L4 hemilaminectomy, spinal cord decompression and IVD fenestration in 1/12 dogs; L6-L7 mini-hemilaminectomy, spinal cord decompression and IVD fenestration in 1/12 dogs; exploratory laparotomy to treat a sublumbar abscess secondary to migrating foreign body in 1/12 dogs; lumbosacral dorsal laminectomy, IVD fenestration and curettage in 5/12 dogs; revision of the previous lumbosacral dorsal laminectomy in 1/12 dogs.

Samples for culture of different tissues were taken in 22/25 (88%) dogs. Urine culture was performed in 14/25 (56%) dogs and was positive in 3 dogs: Proteus mirabilis (*n* = 2) and Escherichia coli (*n* = 1). A total of 13/25 (52%) dogs had blood culture performed, which was positive in 5 dogs: *Staphylococcus species* (*n* = 4) and *Acinetobacter species* (*n* = 1). Culture of ultrasound guided fine needle aspirates of the affected intervertebral disc was performed in 2/25 (8%) dogs and resulted negative. A total of 10/25 (40%) dogs underwent surgical sampling of the affected disc and culture was positive in 5/10 dogs: *Staphylococcus species*. (*n* = 3), *Sphingomonas paucimobilis* (*n* = 1) and *Corynebacterium efficiens* (*n* = 1).

Dogs who had a negative tissue culture, or in which no samples were taken, were presumptively diagnosed with discospondylitis based on a combination of history, clinical presentation, neurological examination findings and diagnostic imaging findings.

The median (95% CI) time between MRI-1 and MRI-2 was 123 (28–860) days. In all dogs, STIR and T2-W sequences were performed during both MRI-1 and MRI-2. However, T1-W sequences were not performed in 8 dogs and 4 dogs during MRI-1 and MRI-2, respectively. Contrast (Gadobutrol 1 mmol/ml, Gadovist, BayerⓇ, Germany) at a dose of 0.1 ml/kg was not administered to 10 out 25 (40%) dogs during MRI-1 and 5 out of 25 (20%) dogs during MRI-2. The presence or absence of specific MRI features detected on MRI-1 and MRI-2 are summarized in Table 2.

At the time of MRI-2, 16 dogs were asymptomatic and 9 were symptomatic. The time between MRI-1 and MRI-2 did not differ (*p* = 0.813) between symptomatic [123(34–620) days] and asymptomatic dogs [136 (28–860) days]. Furthermore, the type of treatment (medical or surgical) following MRI-1 was not different between symptomatic and asymptomatic dogs (Table 3).

**Table 3:** Magnetic resonance imaging (MRI) features detected on initial presentation (MRI-1) and follow-up (MRI-2) in 25 dogs with discospondylitis.

**MRI features**	**MRI-1**	**MRI-2**	**Presence on MRI-1 vs. MRI-2**				
	**(*n*)**	**(*n*)**	**Not in MRI-1-Yes**	**Yes in MRI-1—Not**	**Yes in MRI-1—Yes**	**No in MRI-1—Not**	***p*-value**
			**in MRI-2**	**in MRI-2**	**in MRI-2**	**in MRI-2**	
Paravertebral tissue STIR hyperintensity	20	16	1	5	15	4	0.22
Paravertebral tissue contrast enhancement	14^*^	16^*^	0	3	8	1	0.25
Epidural contrast enhancement	12^*^	15^*^	0	0	8	1	n/a
Epidural STIR hyperintensity	14	11	2	5	9	9	0.45
IVD STIR hyperintensity	15	9	2	8	7	8	0.11
IVD contrast enhancement	12^*^	10^*^	1	3	6	1	0.62
Vertebral endplate T2-W hyperintensity	10	9	2	3	7	13	1
Vertebral endplate T2-W hypointensity	10	7	1	4	6	14	0.37
Vertebral endplate STIR hyperintensity	20	16	0	4	16	5	0.13
Vertebral endplate T1-W hypointensity	13^∇^	15^∇^	3	5	7	3	0.72
Vertebral endplate T1-W eroded	12^∇^	11^∇^	2	2	9	5	0.62
Vertebral endplate T1-W destroyed	3^∇^	5^∇^	2	1	2	13	1
Vertebral endplate T1-W eroded + destroyed	14^∇^	16^∇^	1	0	14	3	1
Length of vertebral body changes >25%	11	8	1	4	7	13	0.37
Vertebral endplate contrast enhancement	14^*^	20^*^	1	0	11	0	n/a
Lymphadenomegaly	13	7	2	7	5	10	0.18
Neural tissue compression	19	15	0	4	15	6	0.13

*n, number of dogs; STIR, short-tau inversion recovery; IVD, intervertebral disc; T2-W, T2-Weighted; T1-W, T1-Weighted; n/a, not applicable*.

* *A total of 15 and 20 dogs were administered contrast during MRI-1 and MRI-2, respectively*.

∇ *A total of 17 and 21 dogs had T1-W sequence performed during MRI-1 and MRI-2, respectively*.

At the time of MRI-2 22/25 dogs were receiving treatment, which consisted of antibiotic-therapy in 10/22 dogs, analgesic therapy in 2/22 dogs and a combination of the two in 10/22. All except three symptomatic cases (7/10) were receiving antibiotics and pain relief at the time or MRI-2, and 1/15 cases was receiving analgesic medication alone. Two (2/15) symptomatic cases were not receiving medication. Thirteen (13/15) asymptomatic dogs were receiving antibiotics, which was combined with analgesic therapy in 4/15 cases. One (1/15) asymptomatic case was not receiving any treatment, and one case was receiving analgesic medication alone (1/15).

Based on MRI-2, 20 out of 25 (80%) and 18 out of 25 (72%) dogs were considered to have presumptive active discospondylitis at the first and second assessment by observer 1, respectively (*p* = 0.62). Observer 2 classified 7/25 (72%) cases as active discospondylitis. The Cohen's kappa (k) coefficient was 0.6 and 0.4 indicating a moderate agreement between the observers on first and second assessment, respectively.

After first assessment of MRI-2 by observer 1, 10/13 medically treated and 10/12 dogs surgically treated dogs were considered to have active discospondylitis (*p* > 0.99). After the second assessment of MRI-2 by observer 1, 8/13 medically treated and 10/12 dogs surgically treated were considered to have active discospondylitis (*p* = 0.38). Following attribution of the classification of active and inactive, it was noted that all dogs with lymphadenomegaly (7/25) on MRI-2 had been attributed an active classification. All dogs with soft tissue STIR hyperintensity on MRI-2 (16/25) and 13/14 dogs with STIR epidural hyperintensity had also been classified as active. A total of 17/20 with STIR hyperintense endplates were also classified as active.

A systematic difference between MRI evaluation of active and inactive discospondylitis in symptomatic and asymptomatic dogs was found on MRI-2 after both assessments by observer 1 (Table 3). At first assessment, the sensitivity and specificity of the association between active and inactive scores and symptomatic or asymptomatic clinical status were 40% (21.88–61.34) and 80% (37.55–98.97), respectively. On the second assessment, the sensitivity was 38.9% (20.31–61.38) and the specificity was 71.4% (35.89–94.92).

The antibiotic therapy was continued in 8/15 asymptomatic dogs, 7 of which were considered to have active disease on MRI-2; antibiotic therapy was not continued in 6/15 asymptomatic dogs, 3 of which were classified as active. One asymptomatic case was euthanised due to unrelated disease. The antibiotic therapy was continued in 8/10 symptomatic dogs, 5 of which were considered to have active disease on MRI-2. One (1/10) symptomatic case was euthanised due to disease progression and one (1/10) was not continued on antibiotic-therapy: both cases had MRI-2 classified as active disease.

A total of 4/25 dogs underwent urine (2/4), intervertebral disc (1/4) and blood cultures (1/4): 2/4 of the cases were symptomatic and 0/4 of the cultures were positive.

From the logistic regression, MRI-2 epidural contrast enhancement (*p* = 0.997), epidural STIR hyperintensity (*p* = 0.997), endplate T2-W hyperintensity (*p* = 0.281), endplate STIR hyperintensity (*p* = 0.998), medical treatment (*p* = 0.392) were included in the final model and were not associated with the clinical status. Hosmer-Lemshow test (*p* = 0.78) and Likelyhood ratio test (*p* = 0.016) confirmed the good fitness of the model used.

## Discussion

This study assessed the MRI-2 findings in a group of dogs presumptively diagnosed with, and treated for, discospondylitis. According to our results, no systematic difference was found between MRI-1 and MRI-2 features. There was a systematic difference between the subjective classification of active and inactive disease on MRI-2 and the presence of clinical signs with a sensitivity and specificity of 38.9–40 and 71.4–80% respectively. Whilst a systematic difference was found, these findings illustrate that “inactive disease” on MRI is associated with asymptomatic cases. However, the low sensitivity illustrates the subjective and likely inaccurate imaging classification of “active disease” which was evenly distributed between the symptomatic and asymptomatic groups. Therefore, routine MRI-2 might not provide useful information in asymptomatic dogs treated for discospondylitis in the clinical setting, as no specific features were associated with the presence or absence of clinical resolution.

Despite the low number of dogs included in this study, the population reflected previous findings (1, 12, 22): discospondylitis affected mainly the lumbosacral IVD of intact male dogs; presenting clinical signs were unspecific but spinal pain was the most frequent; *Staphylococcus species* were the most frequent infectious agents isolated on available tissue samples.

Both MRI-1 and MRI-2 were available to the observers for comparison, as they would be in the clinical setting. Active or inactive discospondylitis scores were attributed based on the presence or absence of a regional inflammatory process, that is commonly used to distinguish a degenerative from an infectious process during the diagnosis of discospondylitis (7, 21–23). However, the authors recognize that the presence of an active infection cannot be discarded purely based on the absence of paravertebral soft tissue changes and regional inflammatory changes (ex: lymphadenomegaly), as resolution of soft tissue infection may precede that of the avascular IVD (24, 25). In the present study, the classification of “inactive” discospondylitis score was mostly attributed to asymptomatic cases. However, this study revealed that while a dog without signs of active disease on MRI-2 is probably asymptomatic, the opposite might not be true: a dog with features interpreted as active discospondylitis on MRI-2 may be symptomatic or asymptomatic, suggesting that presumptive inflammatory changes may be present in the absence of an active infectious process. In addition, asymptomatic dogs were not more likely to have presumptive inactive disease on MRI-2. Especially in clinically improved cases, routine follow up MRI will likely prove challenging to be interpreted and may lead to additional testing or unnecessary interventions. These findings are consistent with previous studies in human medicine in which routine follow-up MRIs in asymptomatic patients were of questionable value (18–20).

While this study was retrospective, interpretation of symptomatic and asymptomatic cases was straight forward in all instances as the clinician clearly specified the clinical status during the request for MRI-2. However, we cannot categorically exclude that some dogs with neural tissue compression secondary to degenerative disease, such as intervertebral disc protrusion, may have been included in the symptomatic group based on the presence of neuropathic pain. Given that neural tissue compression was evenly distributed through the symptomatic and asymptomatic groups, and that the majority of our cases was asymptomatic, it is unlikely that this would have significantly affected the findings of our study. The even distribution of dogs with neural tissue impingement between groups supports the previously reported low association between presence of neural tissue compression and clinical signs in dogs with degenerative spinal disease (26–29).

In the present study, discospondylitis was presumptively diagnosed after 9 to 102 days from spinal surgery in 20% of dogs. To the authors' knowledge, post-operative MRI soft tissue changes have not been described in dogs, and interpretation between the expected normal post-operative inflammation or presence of infection is challenging. In theory, the soft tissue damage and inflammation caused by the surgical approach is foreseeable to cause short- and long-term MRI changes associated with inflammation and fibrosis of the paravertebral tissues, respectively. It is therefore possible that we may have included false-positive cases where the MRI changes were associated with normal post-surgical inflammation and not with an infectious process. However, the presumptive diagnosis of discospondylitis in each case was a combination of history, clinical signs, laboratory data and MRI findings. A study reporting post-operative MRI changes in dogs following lumbosacral dorsal laminectomy described a high frequency of contrast enhancing epidural tissue which suppressed fully on fat saturation sequences (29). In our study, the included post-operative cases were presumptively diagnosed with discospondylitis based on presence of clinical signs indicating neurological deterioration despite previous successful decompressive surgery, absence of neural tissue compression on MRI-1, and changes involving the vertebral endplates and vertebral body beyond the expected following surgery (29). In our study, two dogs developed discospondylitis following L7-S1 dorsal laminectomy, two dogs following thoraco-lumbar hemilaminectomy and one dog following a cervical ventral slot. The soft tissue changes detected on MRI-1 were considered significantly more extensive than expected for a normal post-operative MRI study and, for this reason, they were considered representative of an infectious process (29). Similarly, the dogs treated surgically after MRI-1 could have been a source of bias as the post-surgical soft tissue changes could have been considered a sign of an active discospondylitis process on MRI-2. There was no difference in the proportion of dogs classified with active or inactive discospondylitis on MRI-2 between the treatment groups but it cannot be categorically excluded that a difference may be present with a larger sample size group.

In human medicine, suspected infectious diseases with negative tissue culture, such as spondylodiscitis, vertebral osteomyelitis, sepsis, endocarditis and periprosthetic joint infections, have been widely reported (30–34). In our study, 10 out of 12 dogs in which discospondylitis was treated surgically, had an IVD sample taken. The culture was negative in five of them. Negative culture on canine discospondylitis is not an uncommon finding (5, 9) and the following causative factors should be considered: (1) lack of sensitivity of culture medium to detect all infecting bacteria; (2) different types of infectious organisms (i.e. fungal or parasitic) for which specific cultures were not acquired, (3) prior antibiotic exposure; (4) slow-growing or fastidious bacteria, (5) intracellular bacteria that cannot be cultured with the available methods; (6) sampling error or insufficient sample (30–32). Further studies are needed to understand the driving cause for negative culture on dogs affected with discospondylitis.

Vertebral endplate contrast enhancement is frequently reported in the diagnosis of discospondylitis (7, 21, 22). In the present study, vertebral endplate enhancement was present in all dogs on MRI-2 that received intravenous contrast medium, despite the presence or absence of clinical signs. This finding suggests that vertebral endplate enhancement is likely present in the absence of active infectious disease and may therefore be an unreliable feature when analyzing MRI-2 for evidence of active infection. The underlying reason for the presence of contrast enhancement on a higher proportion of dogs on MRI-2 compared to MRI-1 is unclear. A plausible hypothesis may be the presence of greater vascular supply and increasing granulation tissue associated with healing results in this finding. These findings are in line with previous reports in human literature (18, 20).

In a previous study, a high incidence of contrast enhancement of the vertebral endplates was also reported in reactive, but not infectious, endplate disorders and highlighted that there is overlap between the signal patterns of degenerative, infectious, and neoplastic diseases (21). However, in that study, no dogs had contrast enhancement of the IVD, except if they had discospondylitis. In our study, no difference on IVD contrast enhancement was found between symptomatic and asymptomatic dogs on MRI-2. Intervertebral disc enhancement may, therefore, likely be present in inactive discospondylitis and should therefore be interpreted with caution on follow-up MRI. This finding is further supported by a previous study which reported a high frequency of intervertebral disc enhancement in follow-up MRI in dogs who underwent dorsal lumbosacral laminectomy (29). Neovascularisation of the intervertebral disc during healing is also hypothesized as a potential underlying pathophysiological mechanism for contrast enhancement in cases who have been successfully treated for discospondylitis.

The retrospective nature of this study is a limitation as well as the low number of subjects allocated to the symptomatic and asymptomatic group. This is likely a result of the low incidence of discospondylitis in the general population of dogs (1). Furthermore, as discospondylitis carries an overall good prognosis (1, 12), the ambiguous utility of follow up MRI paired with its challenging interpretation, the need for a general anesthetic, and the associated monetary and emotional implications to the owners, are likely to have been driving causes for the limited number of cases that underwent MRI-2.

The MRI protocols were not standardized and some of the cases did not receive intravenous contrast. This lack of homogeneity likely stems from the fact that once the clinicians involved in the case consider the images diagnostic, they may refrain from performing additional sequences due to monetary restraints, as well as to prevent unnecessary prolonging of anesthesia.

The two observers had a moderate interindividual agreement during the classification of active or inactive MRI-2. The lack of a perfect agreement likely stems from the ambiguity of MRI-2 findings, in part due to the frequent presence of overlapping imaging features which are suspected to represent presence and absence of infection. This finding highlights the importance of combining clinical, laboratory and imaging data during clinical decision making as MRI features alone are subject to individual interpretation. It is possible that the inclusion of a greater number of reviewers or review of serial MRI studies would have yielded different results.

Information regarding patient outcomes following MRI-2, especially in asymptomatic cases, would have been of value to understand if these cases have indeed inactive disease, or if the clinical signs relapsed further down the line indicating persistent infection. Given the retrospective nature of this study and that most dogs were continued on antibiotics despite the classification of “inactive” disease, the ability to draw any conclusions is hindered. Further studies should include a set protocol regarding the length of treatment and interval of time between MRI-1 and MRI-2 so that conclusions can be inferred regarding usefulness of MRI-2 in clinical decision making.

Lastly, considering the influence of field strength on the tissue contrast (35), the changes reported in this study may not be applicable to high field MRI.

In conclusion, this study did not identify meaningful evidence to support routine MRI-2 in dogs treated for discospondylitis and in which clinical signs have resolved. In addition, this study did not identify specific MRI characteristics which are associated with the clinical status of dogs presumptively diagnosed with discospondylitis. The utility of MRI in assessing patients that continue to have clinical signs, and specifically the role of contrast in evaluating these cases, remains unclear at this stage. Considering the small population size, the heterogenicity of the cases and MRI protocols, and the fact that not all dogs received contrast, further studies are required to evaluate the clinical relevance of intervertebral disc and vertebral endplate enhancement on follow-up MRI.

## Data Availability Statement

The raw data supporting the conclusions of this article will be made available by the authors, without undue reservation.

## Ethics Statement

Ethical review and approval was not required for the animal study because no ethical approval was obtained considering the retrospective nature of this cross-sectional study, and prior acquisition of owner consent for patient data to be included in scientific studies. Written informed consent was obtained from the owners for the participation of their animals in this study.

## Author Contributions

MdF, AC, and ES acquired the data. MdF and EV analyzed and interpreted the data. MdF drafter the article. All authors contributed to the conception and design of the study, contributed to manuscript revision, read, and approved the submitted version.

## Conflict of Interest

All authors were employed by company Dick White Referrals, part of Linnaeus Veterinary Ltd. The remaining authors declare that the research was conducted in the absence of any commercial or financial relationships that could be construed as a potential conflict of interest.

## Publisher's Note

All claims expressed in this article are solely those of the authors and do not necessarily represent those of their affiliated organizations, or those of the publisher, the editors and the reviewers. Any product that may be evaluated in this article, or claim that may be made by its manufacturer, is not guaranteed or endorsed by the publisher.

## References

[1] Burkert BA Kerwin SC Hosgood GL Pechman RD Fontenelle JP. Signalment and clinical features of diskospondylitis in dogs: 513 cases (1980-2001). J Am Vet Med Assoc. (2005) 227:268–75. 10.2460/javma.2005.227.268 16047665

[2] De Stefani A Garosi LS McConnell FJ Diaz FJ Dennis R Platt SR. Magnetic resonance imaging features of spinal epidural empyema in five dogs. Vet Radiol Ultrasound. (2008) 49:135–40. 10.1111/j.1740-8261.2008.00339.x 18418993

[3] Hovi I Lamminen A Salonen O Raininko R. MR imaging of the lower spine. Differentiation between infectious and malignant disease. Acta Radiol. (1994) 35:532–40. 10.1080/02841859409173318 7946673

[4] Dagirmanjian A Schils J McHenry MC. MR imaging of spinal infections. Magn Reson Imaging Clin N Am. (1999) 7:525–38. 10.1016/S1064-9689(21)00573-0 10494533

[5] Ruoff CM Kerwin SC Taylor AR. Diagnostic Imaging of Discospondylitis. Vet Clin North Am Small Anim Pract. (2018) 48:85–94. 10.1016/j.cvsm.2017.08.007 28964545

[6] Harris JM Chen AV Tucker RL Mattoon JS. Clinical features and magnetic resonance imaging characteristics of diskospondylitis in dogs: 23 cases (1997-2010). J Am Vet Med Assoc. (2013) 242:359–65. 10.2460/javma.242.3.359 23327179

[7] Carrera I Sullivan M McConnell F Gonçalves R. Magnetic resonance imaging features of discospondylitis in dogs. Vet Radiol Ultrasound. (2011) 52:125–31. 10.1111/j.1740-8261.2010.01756.x 21388462

[8] Cherubini GB Cappello R Lu D Targett M Wessmann A Mantis P. findings in a dog with discospondylitis caused by Bordetella species. J Small Anim Pract. (2004) 45:417–20. 10.1111/j.1748-5827.2004.tb00259.x 15352413

[9] Adamo PF Cherubini GB. Discospondylitis associated with three unreported bacteria in the dog. J Small Anim Pract. (2001) 42:352–5. 10.1111/j.1748-5827.2001.tb02473.x 11480903

[10] Tipold A Stein VM. Inflammatory diseases of the spine in small animals. Vet Clin North Am Small Anim Pract. (2010) 40:871–9. 10.1016/j.cvsm.2010.05.008 20732596

[11] Betbeze C McLaughlin R. Canine diskospondylitis: its etiology, diagnosis, and treatment. Vet Med. (2002) 97:673–8.

[12] Gilmore DR. Lumbosacral diskospondylitis in 21 dogs. J Am Anim Hosp Assoc. (1987) 23:57–61.22505240

[13] Schwartz M Boettcher IC Kramer S Tipold A. Two dogs with iatrogenic discospondylitis caused by meticillin-resistant Staphylococcus aureus. J Small Anim Pract. (2009) 50:201–5. 10.1111/j.1748-5827.2008.00720.x 19320814

[14] Shamir MH Tavor N Aizenberg T. Radiographic findings during recovery from discospondylitis. Vet Radiol Ultrasound. (2001) 42:496–503. 10.1111/j.1740-8261.2001.tb00976.x 11768515

[15] Kraft SL Mussman JM Smith T Biller DS Hoskinson JJ. Magnetic resonance imaging of presumptive lumbosacral discospondylitis in a dog. Vet Radiol Ultrasound. (1998) 39:9–13. 10.1111/j.1740-8261.1998.tb00318.x 9491511

[16] Gonzalo-Orden JM Altonaga JR Orden MA Gonzalo JM. Magnetic resonance, computed tomographic and radiologic findings in a dog with discospondylitis. Vet Radiol Ultrasound. (2000) 41:142–4. 10.1111/j.1740-8261.2000.tb01467.x 10779073

[17] Carragee EJ. The clinical use of magnetic resonance imaging in pyogenic vertebral osteomyelitis. Spine. (1997) 22:780–85. 10.1097/00007632-199704010-00015 9106320

[18] Gillams AR Chaddha B Carter AP. MR appearances of the temporal evolution and resolution of infectious spondylitis. Am J Roentgenol. (1996) 166:903–07. 10.2214/ajr.166.4.8610571 8610571

[19] Veillard E Guggenbuhl P Morcet N Meadeb J Bello S Perdriger A. Prompt regression of paravertebral and epidural abscesses in patients with pyogenic discitis. Sixteen cases evaluated using magnetic resonance imaging. Joint Bone Spine. (2000) 67:219–27. 10.1016/S1169-8330(00)80034-4 10875322

[20] Berbari EF Kanj SS Kowalski TJ Darouiche RO Widmer AF Schmitt SK. 2015 Infectious Diseases Society of America (IDSA) clinical practice guidelines for the diagnosis and treatment of native vertebral osteomyelitis in adults. Clin Infect Dis. (2015) 61:e26–46. 10.1093/cid/civ482 26229122

[21] Gendron K Doherr MG Gavin P Lang J. Magnetic resonance imaging characterization of vertebral endplate changes in the dog. Vet Radiol Ultrasound. (2012) 53:50–6. 10.1111/j.1740-8261.2011.01861.x 21992691

[22] Thomas WB. Diskospondylitis and other vertebral infections. Vet Clin North Am Small Anim Pract. (2000) 30:169–82. 10.1016/S0195-5616(00)50008-4 10680214

[23] Ledermann HP Schweitzer ME Morrison WB Carrino JA. MR imaging findings in spinal infections: rules or myths? Radiology. (2003) 228:506–14. 10.1148/radiol.2282020752 12802004

[24] Daly C Ghosh P Jenkin G Oehme D Goldschlager T. A review of animal models of intervertebral disc degeneration: pathophysiology, regeneration, and translation to the clinic. Biomed Res Int. (2016) 2016:5952165. 10.1155/2016/5952165 27314030 PMC4893450

[25] Jackson AR Eismont A Yu L Li N Gu W Eismont F. Diffusion of antibiotics in intervertebral disc. J Biomech. (2018) 76:259–62. 10.1016/j.jbiomech.2018.06.008 29941209 PMC6082158

[26] Mukherjee M Jones JC Holaskova I Raylman R Meade J. Phenotyping of lumbosacral stenosis in Labrador retrievers using computed tomography. Vet Radiol Ultrasound. (2017) 58:565–80. 10.1111/vru.12520 28691168

[27] Linn LL Bartels KE Rochat MC Payton ME Moore GE. Lumbosacral stenosis in 29 military working dogs: epidemiologic findings and outcome after surgical intervention (1990–1999). Vet Surg. (2003) 32:21–9. 10.1053/jvet.2003.50001 12520486

[28] De Decker S Gielen IM Duchateau L van Bree HJ Waelbers T Bavegems V. Morphometric dimensions of the caudal cervical vertebral column in clinically normal doberman pinschers, english foxhounds and doberman pinschers with clinical signs of disk-associated cervical spondylomyelopathy. Vet J. (2012) 191:52–7. 10.1016/j.tvjl.2010.12.017 21257325

[29] Rapp M Ley CJ Hansson K Sjöström L. Postoperative computed tomography and low-field magnetic resonance imaging findings in dogs with degenerative lumbosacral stenosis treated by dorsal laminectomy. Vet Comp Orthop Traumatol. (2017) 30:143–52. 10.3415/VCOT-16-06-0096 28094419

[30] Tattevin P Watt G Revest M Arvieux C Fournier PE. Update on blood culture-negative endocarditis. Med Mal Infect. (2015) 45:1–8. 10.1016/j.medmal.2014.11.003 25480453

[31] Thorndike J Kollef MH. Culture-negative sepsis. Curr Opin Crit Care. (2020) 26:473–7. 10.1097/MCC.0000000000000751 32773615

[32] Palan J Nolan C Sarantos K Westerman R King R Foguet P. Culture-negative periprosthetic joint infections. EFORT Open Rev. (2019) 4:585–94. 10.1302/2058-5241.4.180067 31754464 PMC6836077

[33] Kasalak Ö Adams HJA Jutte PC Overbosch J Dierckx RAJO Wouthuyzen-Bakker M. Culture yield of repeat percutaneous image-guided biopsy after a negative initial biopsy in suspected spondylodiscitis: a systematic review. Skeletal Radiol. (2018) 47:1327–35. 10.1007/s00256-018-3006-5 29915936 PMC6105158

[34] Dogan M Simsek AT Yilmaz I Karaarslan N. Evaluation of Empirical Antibiotic Treatment in Culture Negative Pyogenic Vertebral Osteomyelitis. Turk Neurosurg. (2019) 29:816–22. 10.5137/1019-5149.JTN.25018-18.2 31049918

[35] Jensen TS Bendix T Kjaer P. Characteristics and natural course of vertebral endplate signal (Modic) changes in the Danish general population. BMC Musculoskelet Disord. (2009) 10:1–9. 10.1186/1471-2474-10-81 19575784 PMC2713204

